# “Apple does not fall far from the tree” – subclinical atherosclerosis in children with familial hypercholesterolemia

**DOI:** 10.1186/s12944-020-01335-2

**Published:** 2020-07-14

**Authors:** Michał Podgórski, Katarzyna Szatko, Małgorzata Stańczyk, Monika Pawlak-Bratkowska, Agnieszka Konopka, Ewa Starostecka, Marcin Tkaczyk, Sebastian Góreczny, Lena Rutkowska, Agnieszka Gach, Maciej Łukaszewski, Piotr Grzelak, Maciej Banach

**Affiliations:** 1 Department of Diagnostic Imaging of Polish Mother’s Memorial Hospital Research Institute, 281/289 Rzgowska Street, 93-338 Lodz, Poland; 2 Department of Pediatrics and Immunology with Nephrology Unit of Polish Mother’s Memorial Hospital Research Institute, Lodz, Poland; 3 Regional Rare Disease Center of Polish Mother’s Memorial Hospital Research Institute, Lodz, Poland; 4 Department of Cardiology of Polish Mother’s Memorial Hospital Research Institute, Lodz, Poland; 5 Department of Genetics, Polish Mother’s Memorial Hospital Research Institute, Lodz, Poland; 6 Department of Hypertension, Medical University of Lodz, Lodz, Poland; 7 Department of Cardiology and Congenital Diseases of Adults, of Polish Mother’s Memorial Hospital Research Institute, Lodz, Poland

**Keywords:** Sonography, Intima-media complex thickness, Tonometry, Arteriosclerosis, Speckle-tracking, Familial hypercholesterolemia, Pediatric patients, Cardiovascular risk

## Abstract

**Background:**

Familial hypercholesterolemia (FH) increases the risk of atherosclerosis in children and adults. Atherosclerotic cardiovascular disease in young patients FH is usually subclinical but recognition of children with more pronounced changes is crucial for adjusting effective management. Aim of this research was to use ultrasonography with two-dimensional speckle tracking (2DST) and tonometry to evaluate atherosclerotic changes in patients with FH (parents and their offspring).

**Methods:**

Applanation tonometry and carotid arteries sonography with evaluation of the intima-media complex thickness (IMCT) and application of the 2DST were performed in 20 families with FH (20 parents and 29 children). The same size control group (age and sex matched) was included. Results were compared between peers and between generations together with the correlation analysis.

**Results:**

Adults with FH, in comparison with healthy peers, presented significantly more atherosclerotic plaques (9 vs. 2, *p* = 0.0230), had significantly thicker IMC (0.84 ± 0.19 vs. 0.56 ± 0.06 mm, *p* < 0.0001) and had stiffer arterial wall (for stain: 6.25 ± 2.3 vs. 8.15 ± 2.46, *p* = 0.0103). In children from both groups there were no atherosclerotic plaques and IMCT did not differ significantly (0.42 ± 0.07 vs. 0.39 ± 0.04, *p* = 0.1722). However, children with FH had significantly stiffer arterial wall according to 2DST (for strain: 9.22 ± 3.4 vs. 11.93 ± 3.11, *p* = 0.0057) and tonometry (for the pulse wave velocity: 4.5 ± 0.64 vs.3.96 ± 0.62, *p* = 0.0047). These parameters correlated with atherosclerosis surrogates in their parents (*p* < 0.001) but were not significantly affected by presence of presumed pathogenic gene variant.

**Conclusions:**

Children with FH presented subclinical atherosclerosis manifested as decreased arterial wall elasticity. Degree of stiffening was associated with advancement of atherosclerosis in their parents but did not present significant association with gene variants. Sonography with application of 2DST seems to be a good candidate for comprehensive evaluation of atherosclerosis in families with FH.

## Introduction

Familial hypercholesterolemia (FH) is an autosomal dominant hereditary disease, causing life-long elevated plasma LDL cholesterol (LDL-C) levels. The homozygous form is rare but recent study suggested that the heterozygous one might affect 1 in 200–250 individuals from the general population [1]. Both, pre-mortem [2] and post-mortem studies [3] confirmed FH association with development of premature atherosclerosis in children. Moreover, atherosclerotic cardiovascular disease (ASCVD) that usually starts in middle age or letter was also reported to progresses rapidly at an age of around 10 years in patients with FH. Hence, guidelines for Europe and America highlight that early diagnosis of FH and statin treatment from childhood are necessary for preventing the early-onset ASCVD [4, 5].

Since atherosclerosis in children with FH typically presents in subclinical stage, standard diagnostic imaging methods might not be sufficient. Even discreet morphological changes (thickening of intima-media complex [IMC] evaluated with ultrasound [6, 7]), may be absent while the function of the arterial wall is impaired already. This stiffening process can be assessed with applanation tonometry, with the pulse wave velocity (PWV) and augmentation index (AI) being its two common surrogates. Although tonometry is stated to be the gold standard technique it is not routinely used in clinical practice [8].

On the other hand, a novel sonographic technique - the 2-dimensional speckle tracking (2DST) - can be an alternative that proved its usefulness in evaluation of risk groups of adults [9, 10] and children as well [11]. This method allows to assess pattern of arterial wall deformation due to the flowing pulse wave. Degree of deformation (expressed as strain in %) and its acceleration in time (expressed as strain rate in 1/s) reflect local arterial wall elasticity [9]. Thus, sonography becomes a comprehensive technique that allows for tailoring the diagnostic method for each family member. Particularly that in children discreet disturbances in arterial wall function can be recognized with 2DST, while in their parents atherosclerotic plaques, including their hemodynamic significance, can be assessed with 2D and Doppler ultrasound.

This study aims to compare morphological and functional surrogates of atherosclerosis between family members with FH and healthy peers. First hypothesis is that in families with FH both parents and their offspring will present significantly more advanced atherosclerosis than their healthy counterparts. Second one, that both diagnostic techniques (ultrasound and applanation tonometry) will allow to recognize functional abnormalities. And finally, that the degree of atherosclerotic changes in children with FH will be associated with their aggravation in parents and with type of mutation leading to FH. Confirmation of these hypotheses will enforce the usage of ultrasound with the 2DST technique as a method of choice in evaluation of atherosclerosis in children and adults with FH.

## Methods

To this cross-sectional study 20 FH families were recruited from the Regional Rare Disease Center in the Polish Mother’s Memorial Hospital Research Institute (PMMHRI). They comprised 20 parents (10 males, and 10 females) and their 29 children (13 males, and 16 females). The FH was confirmed based on genetic array and/or the Dutch Lipid Clinic Network (DLCN) and Simone Broome Register. In all children the next generation sequencing was performed (MiniSeq, Illumina, Inc., San Diego, US) using custom panel of 21 genes. Obtained data were analyzed with available databases and predictive programs (sorting intolerant from tolerant [SIFT] and polymorphism phenotyping [PolyPhen]). Detected variants were confirmed with the Sanger sequencing. All further examinations were performed within 6 months from the diagnosis of FH in children. Family members not affected with FH were not included. The same number of healthy families (no significant difference in sex and age) was recruited to the control group. Lipid profile of children from the control group was within normal limits while adult Individuals did not present significant abnormalities (levels of total cholesterol (TC), low-density lipoprotein (LDL) and triglycerides (TG) were up to borderline/borderline high levels). Eight adults from the control group reported regular treatment with statins.

In both groups exclusion criteria were chronic diseases (except for hypertension) that might increase the risk of atherosclerosis (i.e.: diabetes mellitus, chronic inflammatory diseases like rheumatoid arthritis or non-specific inflammatory bowel diseases). Hypertension is a common disease, thus it was included into analysis as a risk factor instead of excluding patients from the study. If the exclusion criterion was present in one of family members to be examined the whole family was excluded from the study. No participant had former cardiovascular events (i.e.: acute coronary syndrome or stroke) that were also assumed as criteria excluding the whole family from the study. In both groups data regarding hypertension (diagnosed according to the European Society of Cardiology (ESC) and the European Society of Hypertension (ESH) guidelines as Systolic pressure > 140 mmHg and/or diastolic pressure > 90 mmHg [10.1093/eurheartj/ehy339]) and lifetime tobacco exposure (pack years) were obtained as one of the main atherosclerosis risk factors.

### Study protocol

In all patients blood samples were obtained up to 7 days prior to the further examinations. Lipid profile (TC, LDL, TG, high-density lipoprotein [HDL] and non-HDL cholesterol) and HbA1C % were assessed.

At appointed date family members arrived at the hospital at morning hours fasting. They were measured and weighted in light clothing and without shoes to calculate body mass index (BMI). Then the sonographic and tonometric tests were performed by two independent researches blinded to the group affiliation. Examinations techniques were standard, and the detailed description together with accuracy and reproducibility for adults and children was reported before [9–11].

Briefly, the sonographic examination was performed with the Samsung RS80 apparatus equipped with a high-frequency linear probe (L3-12A) and Arterial Analysis™ software. The ECG trace was obtained, and the blood pressure was measured in patients lying supine. Major atherosclerotic changes were assessed first (atherosclerotic plaques – soft or calcified, focal IMC thickenings of more than 50% of the adjacent parts of the IMC layer [9]).

Then, the B-mode cine loops of the long and short axis through the common carotid artery (CCA) (just below the bulb) were stored during three consecutive heartbeats for each cross-section. If there was no plaque IMCT was assessed (semi-automatically, during end diastole, at the distance of 150–250 points, with the quality index > 0.9). To evaluate strain and strain rate in short axis cine loop the circular region of interest (ROI) was pointed along the border between the wall and arterial lumen. Parameters were assessed automatically and mean results from 6 heartbeats (three for each side) were included into the analysis.

The PWV and AI were evaluated by another researcher (9 years of experience in tonometric studies) using a SphygmoCor applanation tonometer (SphygmoCor, AtCor Medical, New South Wales, Australia). The AI was assessed based on two measurements of the brachial artery pressure. Then, in patients laying supine, the PWV was measured by recording the arterial pressure waveform at the carotid and femoral artery sampling sites. If the operator index was lower that 75% measurements were repeated.

### Statistical analysis

Categorical variables were presented as number and percentages, while continuous variables as mean and standard deviation (SD). In comparison of nominal data between groups the Chi^2^ test was applied. Normality of continues variables distribution was evaluated with the Shapiro-Wilk test. Due to distribution other than normal comparisons of means between two independent subgroups (adults with FH vs. healthy adults and children with FH vs. healthy children) were performed with the Mann-Whitney test. When subgroups were related (adults with FH vs. children with FH and healthy adults vs. healthy children) the Wilcoxon signed rank test was applied. Associations between continuous variables were evaluated with the Spearman’s rank correlation test. The analysis was performed with Statistica 12 software (StatSoft Poland, Cracow, Poland). In general a *p*-value lower than 0.05 was considered significant, however for multiple comparisons the Bonferroni correction was applied.

## Results

Comparison of demographic data and laboratory tests are presented in Table 1. Individuals with FH did not differ significantly according to determined additional risk factors of atherosclerosis (BMI, hypertension, tobacco exposure, increased concentration of HbA1C). However, both parents and children with FH presented significantly higher values of TC, LDL-C, non-HDL-C and TG in comparison to their healthy counterparts, despite the fact that all adults were treated with statins.

**Table 1:** Clinical characteristics of the patients

Feature	Adults with FH	Healthy adults	*P*	Children with FH	Healthy children	*P*
Age [years (SD)]	37.5 (7.4)	38.6 (6.4)	0.5772	11.1 (4.5)	9.8 (4.0)	0.3148
Sex						
Females [n (%)]	10	10	1.000	16	15	0.7924
Males [n (%)]	10	10		13	14	
BMI [kg/m2 (SD)]	24.2 (1.9)	23.9 (1.8)	0.5778	18.5 (3.2)	17.2 (2.1)	0.1087
Smoking (pack years) [years]	6.26 (2.8)	6.28 (4.0)	0.9929	0	0	1.000
Hypertension [n (%)]	7 (35)	6 (30)	0.7924	0	0	1.000
HbA1C	4.89 (0.38)	5.01 (0.15)	0.1523	3.99 (1.29)	4.23 (1.18)	0.8473
TC	316.55 (65.57)	158.52 (16.81)	< 0.0001^*^	365.38 (99.23)	107.30 (18.09)	< 0.0001*
LDL	217.50 (53.47)	80.70 (16.97)	< 0.0001*	242.45 (58.33)	57.00 (12.15)	< 0.0001*
HDL	68.91 (32.32)	58.17 (18.67)	0.1772	48.73 (14.82)	52.30 (8.35)	0.3128
Non-HDL	247.63 (79.82)	100.35 (21.07)	< 0.0001	316.65 (98.05)	55.00 (18.70)	< 0.0001
TG	111.63 (25.21)	93.04 (16.88)	0.0056*	99.35 (32.22)	59.78 (14.33)	< 0.0001*

*HbA1C* Glycosylated Hemoglobin, Type A1C, *TC* total cholesterol, *LDL* Low-density lipoprotein, *HDL* High-density lipoprotein, *TG* Triglycerides, *P p*-value

*Significant differences according to Mann-Whitney test

### Morphological features of atherosclerosis

Adults with FH had significantly more atherosclerotic plaques (total number of 7, mean thickness 4 mm ± 1,2 mm) than parents from the control group (total number of 2, thickness of 1,8 mm and 2,3 mm). In analysis of IMCT adults with FH presented significantly thicker layer than observed in healthy adults (Table 2). Moreover, in both groups parents had significantly thicker IMC than their children (Table 2). In children there were no atherosclerotic plaques and the IMCT did not differ significantly between individuals with FH and healthy one.

**Table. 2:** Comparison of atherosclerosis surrogates between patients with FH and healthy control according to generations

Feature	Adults with FH	Healthy adults	*P*	Children with FH	Healthy children	*P*	Adults with FH	Children with FH	*P*	Healthy adults	Healthy children	*P*
Atherosclerotic plaques [n (%)]	9	2	0.0231	0	0	1.000						
Mean IMCT [mm (SD)]	0.84 (0.19)	0.56 (0.06)	< 0.0001	0.42 (0.07)	0.39 (0.04)	0.1722	0.84 (0.19)	0.42 (0.07)	< 0.0001	0.56 (0.06)	0.39 (0.04)	< 0.0001
PWV [m/s (SD)]	6.12 (1.08)	5.04 (1.38)	0.0055	4.50 (0.64)	3.96 (0.62)	0.0047	6.12 (1.08)	4.50 (0.64)	< 0.0001	5.04 (1.38)	3.96 (0.62)	0.0014
AI [% (SD)]	12.27 (5.01)	16.03 (5.24)	0.0179	18.30 (8.07)	24.43 (7.34)	0.0080	12.27 (5.01)	18.30 (8.07)	0.0039	16.03 (5.24)	24.43 (7.34)	0.0001
Strain [% (SD)]	6.25 (2.30)	8.15 (2.46)	0.0103	9.22 (3.40)	11.93 (3.11)	0.0057	6.25 (2.30)	9.22 (3.40)	0.0011	8.15 (2.46)	11.93 (3.11)	< 0.0001
Strain rate [1/s (SD)]	0.61 (0.27)	0.84 (0.29)	0.0081	0.93 (0.32)	1.25 (0.41)	0.0031	0.61 (0.27)	0.93 (0.32)	0.0006	0.84 (0.29)	1.25 (0.41)	0.0003

*IMCT* intima media complex thickness, *PVW* pulse wave velocity, *AI* augmentation index, *P p*-value

### Functional features of atherosclerosis

**Fig. 1:**
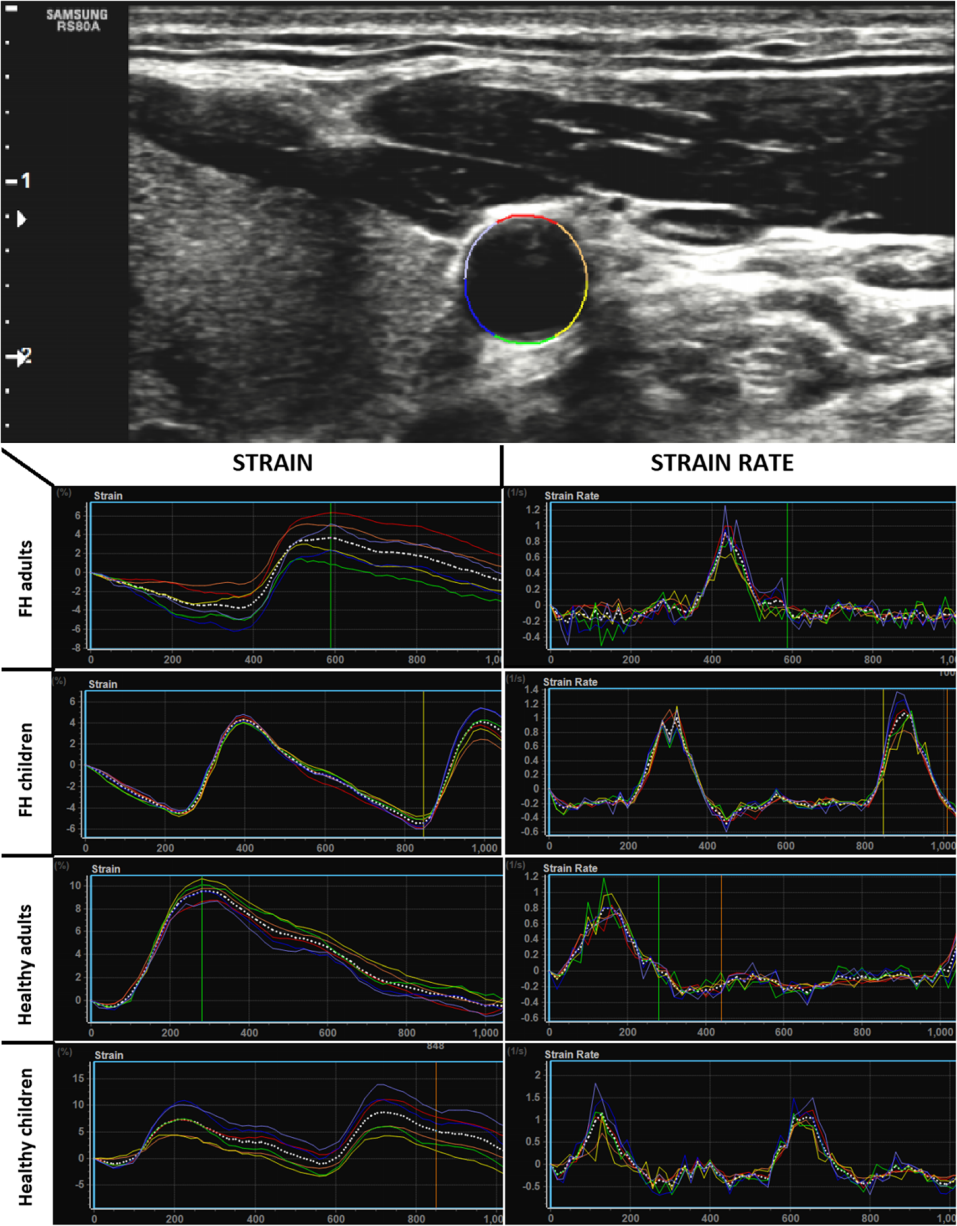
Examples of graphs presenting strain and strain rate in representative individuals from each group of patients. Colored lines represent segments of vessel while dotted white line is a mean value. At the top of the figure there is a grayscale transverse ultrasound of the neck with ROI in the CCA

Both, parents and children with FH, presented significantly increased arterial stiffness in comparison with their healthy counterparts (Table 2). It was confirmed with tonometry as well as with the 2DST (Fig. 1). Stiffness parameters differed also between parents and children from the same group (adults with FH vs. children with FH and healthy adults vs. healthy children, Table 2).

### Association of atherosclerosis markers with gens

In 5 children we found only single, likely pathogenic, gene variant, 14 children had 2 gene variants, 8 children had 3 variant gens, 3 children had 4 gene variants and in two children we did not find any variant from the analyzed panel. Sequencing identified *LDLR* mutations as the most common cause of FH in the tested group. In 17 patients 15 substitutions, one intragenic deletion and one intragenic duplication of *LDLR* were found. Variants were also revealed in *APOE* (6 children), *PCSK9* (4 children), *LCAT* (4 children), *ABCG5* (4 children), *ABCA1* (3 children), *GPIHBP1* (2 children), *SCAP* (2 children) and *APOA* (1 child).

We compared atherosclerosis markers between individuals in whom variants were presumed pathogenic according to prognostic programs or literature. There was only a trend towards more advanced atherosclerotic changes in children with these variants but the difference was not significant (Table 3**)**.

**Table 3:** Comparison of atherosclerosis surrogates between children with presumed pathogenic gene variants and those with presumed benign variants

Feature	Presumed pathogenic variants	Presumed benign variants	*P*
Mean IMCT [mm (SD)]	0.85 (0.22)	0.82 (0.16)	0.6548
PWV [m/s (SD)]	6.36 (1.10)	5.79 (1.01)	0.2304
AI [% (SD)]	11.98 (5.43)	12.69 (4.63)	0.7518
Strain [% (SD)]	5.63 (2.29)	7.13 (2.11)	0.1347
Strain rate [1/s (SD)]	0.58 (0.32)	0.64 (0.20)	0.6283

*IMCT* intima media complex thickness, *PVW* pulse wave velocity, *AI* augmentation index, *P p*-value

### Parameters correlations

In both groups and for both generations there was a significant correlation between IMCT and parameters derived from tonometry and those from 2DST. Only in adults stiffness parameters correlated with IMCT. On the contrary, only in adults with FHs there was a significant correlation between IMCT and stiffness parameters with stiffness parameters in their children (Table 4). In all subgroups, there was no significant association between concentrations of lipid profile components and analyzed surrogates of atherosclerosis.

**Table 4:** Correlations between atherosclerosis surrogates (cumulated data for all subgroups and between parents and children with FH.)

Parameters	Strain				Strain Rate			
	R		*P*		R		*P*	
IMCT	−0.7043		0.0001		−0.6659		0.0001	
PWV	−0.8080		0.0001		−0.7788		0.0001	
AI	0.8793		0.0001		0.8229		0.0001	
Parameters	Children with FH							
	PWV		AI		Strain		Strain Rate	
	R	*P*	R	*P*	R	*P*	R	*P*
Adults with FH								
IMCT	0.5427	0.0091	−0.6520	0.0010	−0.6055	0.0028	−0.6702	0.0006
PWV	0.6497	0.0011	−0.7891	< 0.0001	−0.6970	0.0003	−0.7113	0.0002
AI	−0.5629	0.0064	0.6650	0.0007	0.5739	0.0052	0.6231	0.0020
Strain	−0.5964	0.0034	0.7482	0.0001	0.6374	0.0014	0.7182	0.0002
Strain Rate	−0.6551	0.0009	0.8113	< 0.0001	0.7248	0.0001	0.7767	< 0.0001

*IMCT* intima media complex thickness, *PVW* pulse wave velocity, *AI* augmentation index, *P p*-value

## Discussion

In this study family members with FH, parents as well as their offspring, presented more advanced atherosclerosis than their healthy peers. Adults with FH had both morphological and functional changes while in children with FH only arterial function was impaired (decreased elasticity). Moreover, in patients with FH there was a correlation of atherosclerosis surrogates between adults and their children. On the other hand, presence of presumed pathogenic gene variant in children did not result in significantly aggravated markers of atherosclerosis. Finally, disturbances of arterial wall elasticity were recognized in ultrasound and applanation tonometry and they correlated with each other. However, sonography allows for more comprehensive evaluation of atherosclerosis aggravation.

FH increases the risk of atherosclerosis and predispose to cardiovascular events in younger age than in healthy population [12, 13]. Although monozygotic form is rare, as many as 1 per 250 people from general population is a heterozygote [14]. It gives approximately 4.5 million individuals in Europe, of whom 20–25% are children and adolescents [15]. Despite analyses proofing cost effectiveness of FH screening [16] and treatment standards, which are developed (diet, controlling risk factors, statins [5]) or developing (gene- and cell-based therapies [17]), under-diagnosing of the disease is a major problem [18]. This work provides another argument for introducing screening programs because, even in children with no significant morphological changes, arterial wall function can be already impaired. Diagnosis of subclinical arterial wall stiffening might improve risk stratification and clinical management in FH patients [13, 19].

Many researchers have already confirmed increased risk of atherosclerosis (also subclinical [20]) and subsequent cardiovascular events in adults with FH [21]. Studies concerning children with FH are not as numerous [15, 22–24] but it is well documented that FH children have significantly increased IMCT in carotid arteries, femoral arteries and aorta when compared with healthy children as well as healthy siblings [24, 25]. Although, there are discrepancies in age from which these differences become significant (between 10 and 12 for siblings [24, 26] and 9–11 years for nonrelated controls [22, 25]) and they can also be affected by treatment with statins. Luirink et al., in their study with 20-years long follow up, proofed that although children with FH had initially significantly thicker IMC than healthy siblings, differences become insignificant during years of treatment with statins. It is worth to notice that in aforementioned studies morphological changes were slight (fractions of millimeter). Thus, to reach the level of statistical significance study populations had to be larger than in our research. It may explain why we observed only a slight numerical trend towards thicker IMC. On the other hand, in our group we were able to detect significant deterioration of arterial wall function. It might suggest superiority of arterial wall elasticity surrogates as markers of atherosclerosis in children with FH.

Arterial wall function was reported to be impaired in children with FH, however in former researches it was proofed mainly by flow-mediated dilation (FMD) test [27, 28] and markers calculated from 2D images (e.g. young elastic modulus, beta-stiffness index) [28, 29]. Riggio at al [29]. investigating the group of 44 children with increased cholesterol levels (18 with FH) showed that arterial wall function was impaired in comparison with healthy control, while there was no significant difference in IMCT. Although, they also used echo-tracking software, it was applied to calculate arterial wall elasticity surrogates (beta-stiffness index, arterial compliance, AIx, local PWV, Young elastic modulus) and results were not confirmed by a gold standard method (tonometry). Aggoun et al. [28] using sonography and FMD test also showed that in children with FH (30 males, mean age of 11 ± 2 years old) the arterial wall function was impaired when no significant changes in IMCT were observed. Despite applying surrogates depending on blood pressure in this last study, both studies are in line with our observations, and confirm that there is a continuum of atherosclerotic changes that impairs the arterial wall function first and then affects its morphology.

To conclude the above, in our opinion application of sonography with 2DST technique is more convenient and efficient than employing other methods for evaluation of atherosclerosis. On the contrary to the FMD procedure it does not require several minutes of painful forearm compression. Currently, purchasing of 2DST software is cheaper than buying an applanation tonometer, which handling requires experience. Moreover, repeatability of the stain evaluation with 2DST is higher than reported for tonometry, while their results correlate with each other [11, 30]. Finally, sonography is the most comprehensive technique because after storing a few loops of arterial wall motion not only function surrogates can be calculated in a semiautomatic manner, but also IMCT and atherosclerotic plaques can be assessed, what would be especially important in older patients. To confirm presented opinion, with application of discussed technique it was possible to examine two generations of patients and showed not only that individuals with FH had significantly advanced atherosclerosis than their healthy peers, but also that there was a relation between degree of atherosclerotic changes between parents and their offspring. This last observation is unique for such a young group of patients. Till now, it was only reported that maternal or paternal origin of FH does not affect carotid IMCT or phenotype of plasma lipid levels, while type of transferred mutation does [31, 32]. As far as a direct relation is concerned, in study of 154 families with other cardiovascular risk factors than FH, there was a IMCT correlation between parents and children, however median of age for parents was 61 years and for children 36 years [33].

Recognition of genetic background of the FH becomes important nowadays because particular mutations were reported to affect phenotype of patients and due to new possibilities of treatment [33–37]. Unfortunately, no significant association between presence of presumed pathogenic gene variants and aggravation of atherosclerosis was observed. It might be due to small analyzed group of children and young age, that did not allow to develop differences in phenotype. On the other hand, even in children genetic variability may play a significant role what is suggested a significant correlations of atherosclerosis markers between parents and their offspring who share the same gene variants.

### Strength and study limitation

The main strength of this study is that results obtained based on the novel sonographic technique were confirmed with a gold standard method. Moreover, due to including family members from two generations it was possible to proof comprehensiveness of sonography with 2DST in evaluation of atherosclerosis in adults and children.

Nevertheless, this study has some limitations. Healthy family members were excluded from the study. In comparison of FH children with their healthy siblings significant differences in IMCT were observed from age 7 years. It would be interesting to compare strain and strain rate between them. However, this study addressed relation between adults and children affected with FH. Furthermore, evaluated group did not allow for such an analysis due to not enough of siblings appropriate to enter the study.

Secondly, small study population did not allow to draw conclusions on association between genetic variance and aggravation of atherosclerosis. Nevertheless, our main aim was to indicate convenient and reliable technique for examining whole families with FH what gives foundation for future studies of larger population.

## Conclusions

Children with FH presented subclinical atherosclerosis that manifested with arterial wall stiffening. Aggravation of changes was associated with advancement of atherosclerosis in their parents but was not significantly affected by a type of recognized gene variant. Sonography with application of 2DST seems to be the best candidate for comprehensive evaluation of atherosclerosis in families with FH.

## Data Availability

On request.

## Abbreviations

2DST: Two-dimensional speckle tracking
AI: Augmentation index
ASCVD: Atherosclerotic cardiovascular disease
BMI: Body mass index
DLCN: The Dutch Lipid Clinic Network
ECG: Electrocardiogram
ESH: The European Society of Hypertension
ESC: The European Society of Cardiology
FH: Familial hypercholesterolemia
FMD: Flow-mediated dilation
HbA1C: Glycated hemoglobin
HDL: High-density lipoprotein
IMCT: Intima-media complex thickness
IMC: Intima-media complex
LDL: Low-density lipoprotein
LDL-C: LDL cholesterol
PMMHRI: The Polish Mother’s Memorial Hospital Research Institute
PolyPhen: Polymorphism phenotyping
PWV: Pulse wave velocity
ROI: Region of interest
SIFT: Sorting intolerant from tolerant
SD: Standard deviation
TC: Total cholesterol
TG: Triglycerides
